# Protocol for Objective Measurement of Infants’ Physical Activity using Accelerometry

**DOI:** 10.1249/MSS.0000000000001512

**Published:** 2017-12-02

**Authors:** LUIZA ISNARDI CARDOSO RICARDO, INÁCIO CROCHEMORE MOHNSAM DA SILVA, RAFAELA COSTA MARTINS, ANDREA WENDT, HELEN GONÇALVES, PEDRO RODRIGUES CURI HALLAL, FERNANDO CÉSAR WEHRMEISTER

**Affiliations:** Post-Graduate Program in Epidemiology, Federal University of Pelotas, Pelotas, BRAZIL

**Keywords:** FEASIBILITY STUDIES, MOTION SENSORS, ACCELEROMETER, ACTIGRAPH GT3X+, CHILDREN, MOTOR ACTIVITY

## Abstract

**Purpose:**

This study aimed to evaluate methods for infants’ physical activity measurement based on accelerometry, including the minimum number of measurement days and placement of a wrist or ankle device. We also evaluated the acceptability of the device among infants and mothers.

**Methods:**

A cross-sectional mixed-methods study was conducted on a convenience sample of 90 infants. Physical activity was measured using the Actigraph GT3X+ accelerometer placed on the wrist and/or ankle for 7 consecutive days (worn for 24 h), and a qualitative interview was performed to verify acceptability. The intraclass correlation coefficient (ICC) method and the Bland and Altman’s dispersion diagram were used to verify the minimum number of measurement days. All analyses were stratified by walking status.

**Results:**

The mean (SD) age was 12.9 (1.70) months; the mean acceleration varied between 25.8 m*g* (95% confidence interval (CI), 14.3–52.7) and 27.3 m*g* (95% CI, 17.9–44.5) using the wrist placement, and between 24.9 m*g* (95% CI, 10.6–48.4) and 26.2 m*g* (95% CI, 11.7–65.6) using the ankle placement. The ICC results showed a lower acceleration variability between days among infants incapable of walking; they achieved an ICC of 0.80 with 1 d of measurement in both placements. Among those capable of walking, the minimum number of days to achieve an ICC of 0.80 was 2 d measured at the wrist (0.85; 95% CI, 0.71–0.93) and 3 d measured at the ankle (0.92; 95% CI, 0.84–0.96). The qualitative results pointed to the wrist placement as the preferred placement among the overall sample.

**Conclusions:**

Two and three measurement days with the accelerometer placed on the wrist and ankle, respectively, seemed to adequately represent a week of measurement. The accelerometer placed on the wrist had better acceptance by the infants and mothers.

## Abstract 2

Supplemental digital content is available in the text.

Health benefits attributed to physical activity among adults and adolescents are well established (1,2). Among young children, however, the scenario is different. Despite evidence pointing to the effects of physical activity on present and future health, regarding adiposity, bone health, motor skill development, psychosocial health, cognitive development, and aspects of cardiometabolic health, it is widely assumed that infants and toddlers are already sufficiently physically active and therefore do not require study or intervention (3). Because of this, the available literature regarding the physical activity of young children is scarce, and well-designed studies and accurate measures are still required (4,5).

In this context, an important challenge is how to measure physical activity with such a young sample. Parent reports (based on questionnaires or diaries) are the most widely used method, because of the ease of application and lower cost (6). However, parent reports have a low agreement with objective measures (such as accelerometer and pedometer), which is mainly due to an overestimation of the measure (7,8). Direct observation and pedometers are also feasible alternatives, although the infants’ motor characteristics may not be compatible with the use of pedometers, and the possibility of reactivity should be taken into account when using direct observation (9).

The use of motion sensors, especially accelerometers, has been established as a viable physical activity measurement in young children, because it is an objective measure of body movement and therefore less prone to bias compared with subjective measures (9). Although the recent literature has provided evidence regarding the accuracy and consistency of accelerometers to measure infants’ physical activity, there are limited interpretation and lack of comparison among study protocols, and therefore, there is still a need for feasibility, validity, and reliability assessments, especially among infants and toddlers (5,10). The variations in minimum period of use and placement of the device, as well as thresholds for different physical activity intensities and other general programming options of each device, are factors that undermine the comparability between studies (11–13). Thus, there is a need to discuss the most appropriate methods regarding physical activity measurement among young children using accelerometry, not only by verifying accuracy of the measures but also by considering the acceptability of the device in the infants’ daily life.

Therefore, the present study aims to determine the most appropriate methods for physical activity measurement among infants on the basis of accelerometry, including the minimum number of days of measurement required, placement of the device on the wrist or ankle, and acceptability of the device.

## METHODS

A cross-sectional mixed-methods study with quantitative and qualitative approaches was conducted between October and December 2015. The sampling process was performed by convenience and included children between the ages of 9 and 15 months, to guarantee greater variability in relation to the motor development of the sample. Participants were divided into three groups to ensure heterogeneity regarding socioeconomic status and daily routine in daycare settings or at home. Thus, the sample consisted of 90 infants allocated to one of three groups: 30 infants were enrolled in public daycare, 30 were enrolled in private daycare, and 30 infants did not attend daycare. In each group, 10 infants used the accelerometer on the wrist, 10 on the ankle, and 10 on both wrist and ankle.

Physical activity was measured using the Actigraph GT3X+ accelerometer, for 7 consecutive days; it was worn 24 h·d^−1^ and programmed to initiate capturing 1 h after device placement, with a sample frequency of 60 Hz. The bracelet used to fix the accelerometer was disposable and made of waterproof vinyl. This material does not cause contact dermatitis and is widely used in the manufacture of surgical gloves (14). The inner part of the bracelet was made with a white color to make it less susceptible for the development of allergies by colorants. All decisions regarding the bracelet material were established with a dermatologist specialized in the area. In the event of a dermatological complaint, we had a specialist available to provide treatment; however, no infant needed an appointment during the field work.

During this 7-d period, two telephone calls—after 24 h and after 4 d—were performed to verify the child and mother’s acceptance. The exposure variables were collected from the parents/guardians using face-to-face questionnaires. The exposure variables were the infants’ sex, age in months, maternal age in years (divided into three groups: 17–26, 27–32, and 33–41 yr), and socioeconomic level (tertiles), calculated through an asset index based on principle components analysis, obtained by a socioeconomic standardized questionnaire (15).

For the qualitative section, an open-question–guided interview was performed with the infants’ mothers or legal guardians, conducted and recorded by the study author. The interview focused on the infants’ routine and acceptability of the device, as well as the mother’s perception regarding the best placement and the main concerns in using the accelerometer in such a young child. All interviews were transcribed by a researcher who was not involved in the interviewing process. The responses were grouped according to the respondents’ answers, identifying the most relevant topics to the research questions, and were mainly concerned with the comfort and acceptance of the device by the infants and their caregivers for the different locations of wrist and ankle. The analyses were descriptive, highlighting the reported speech of the interviewees.

The accelerometer data were analyzed in raw form, that is, the acceleration data of the body movement expressed in milligrams (gravitational equivalent: 1000 m*g* = *g* = 9.81 m·s^−2^). The data were analyzed with R-package GGIR (http://cran.r-project.org) in its continuous form, providing the average daily acceleration as an estimate of the total volume of movement or physical activity. The detailed signal processing scheme included the following steps: verification of sensor calibration error using local gravity as a reference (16), detection of sustained abnormally high values, nonwear detection, imputation of invalid data segments by the average of similar time-of-day data points on different days of the measurement, and calculation of the vector magnitude of activity-related acceleration using the Euclidian Norm minus *g* with any negative values rounded up to zero (16).

For analytical purposes, data from the first day of use were excluded because of potential reactivity and also because the first day usually was not a complete measurement day. Potential reactivity was reported in the mother’s qualitative interviews and observed by the high activity level of this first day partially assessed (i.e., <24 h) in comparison with the remaining days in a preliminary count-based analysis (see Figure, Supplemental Digital Content 1, preliminary analysis presenting average daily vector magnitude counts per minute (CPM) and average measurement valid minutes in each placement of the accelerometer, http://links.lww.com/MSS/B116). Furthermore, because the data capturing was initiated 1 h after device placement, the GGIR package does not consider it as a valid day (which is a minimum of 16 complete hours). Because the first day was excluded, the second valid day was established as day 1. Therefore, to establish the minimum days of measurement that could be representative of a week, the mean acceleration of different periods (e.g., 1, 2, 4, 4, and 5 valid days) was compared with the measurement of 6 d (considered as the criterion measure), using the intraclass correlation coefficient (ICC) method. There was a significant difference between the mean weekly acceleration and walking status (wrist: not walking 24.7 ± 3.7 m*g*, walking 28.3 ± 4.5 m*g*, *P* = 0.006; ankle: not walking 20.8 ± 4.8 m*g*, walking 28.4 ± 7.2 m*g*, *P* < 0.001), all analyses were performed stratified for capable or incapable of walking. To determine the minimum number of measurement days to represent the 6-d criterion measure, the acceptable level of agreement between the measures considered was an ICC of 0.80 (17–19).

In addition, descriptive analyses were performed according to the dispersion diagram proposed by Bland and Altman (20) to compare the mean acceleration of the criterion measure with the minimum number of measurement days of each placement (wrist and ankle) established by the ICC analyses. For supplementary material, we provide the Bland–Altman plots comparing the remaining days and the criterion of 6 d in both placements. See Figure, Supplemental Digital Content 2, Bland–Altman plot of the difference between the mean acceleration of 6 and 1 measurement days with the accelerometer placed on the wrist, http://links.lww.com/MSS/B117; Figure, Supplemental Digital Content 3, Bland–Altman plot of the difference between the mean acceleration of 6 and 3 measurement days with the accelerometer placed on the wrist, http://links.lww.com/MSS/B118; Figure, Supplemental Digital Content 4, Bland–Altman plot of the difference between the mean acceleration of 6 and 4 measurement days with the accelerometer placed on the wrist, http://links.lww.com/MSS/B119; Figure, Supplemental Digital Content 5, Bland–Altman plot of the difference between the mean acceleration of 6 and 5 measurement days with the accelerometer placed on the wrist, http://links.lww.com/MSS/B120; Figure, Supplemental Digital Content 6, Bland–Altman plot of the difference between the mean acceleration of 6 and 1 measurement days with the accelerometer placed on the ankle, http://links.lww.com/MSS/B121; Figure, Supplemental Digital Content 7, Bland–Altman plot of the difference between the mean acceleration of 6 and 2 measurement days with the accelerometer placed on the ankle, http://links.lww.com/MSS/B122; Figure, Supplemental Digital Content 8, Bland–Altman plot of the difference between the mean acceleration of 6 and 4 measurement days with the accelerometer placed on the ankle, http://links.lww.com/MSS/B123; and Figure, Supplemental Digital Content 9, Bland–Altman plot of the difference between the mean acceleration of 6 and 5 measurement days with the accelerometer placed on the ankle, http://links.lww.com/MSS/B124. Furthermore, to illustrate the difference between the wrist and the ankle, a Bland–Altman plot was performed comparing the mean acceleration of 6 measurement days between both placements; this was restricted to infants who used the device in both placements simultaneously. Statistical analyses were carried out in the statistical program Stata 12.0.

Furthermore, although the number of individuals in the sample was 90 (30 in each group), the data for the “both placements” group were analyzed in both the wrist and the ankle; therefore, all ICC analyses considered 120 observations (60 in the wrist and 60 in the ankle placement). In addition, the Bland–Altman plots followed the same logic, except for the comparisons between placements which were restricted to those who wore the device in both placements, considering the measurement days as an analytical unit, so the number of observations was 180.

The study was approved by the Ethics Committee of the Superior School of Physical Education of the Federal University of Pelotas, under protocol number 1.178.846. A written consent document was signed by each parent or caregiver before data collection. An authorization from the City Education Department was also obtained to access public daycare facilities.

## RESULTS

### 

#### Quantitative approach

**FIGURE 1:**
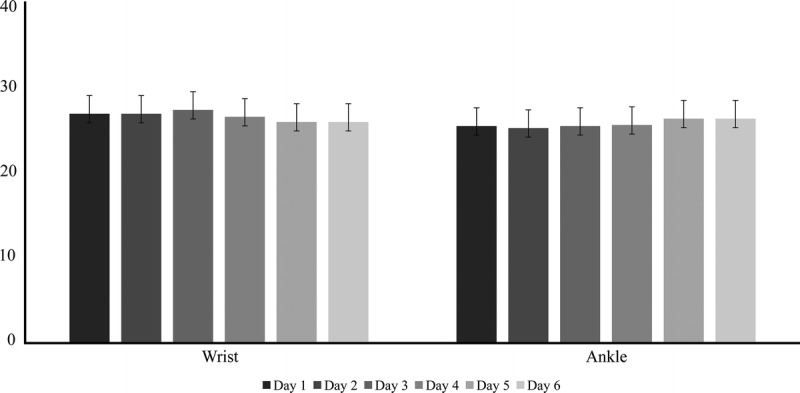
Daily mean acceleration per placement of the accelerometer (Pelotas, RS, Brazil; *N* = 90).

The sample description regarding the characteristics of the infants and mothers is presented in Table 1. The sample was composed of 90 infants, with a mean (SD) age of 12.9 (1.70) months. Furthermore, the sample presents a clear heterogeneity in terms of socioeconomic level and maternal age. Figure 1 shows the daily mean acceleration per placement of the accelerometer. The overall daily mean acceleration varied between 25.8 m*g* (95% confidence interval (CI), 14.3–52.7) and 27.3 m*g* (95% CI, 17.9–44.5) when the accelerometers were placed on the wrist, and between 24.9 m*g* (95% CI, 10.6–48.4) and 26.2 m*g* (95% CI, 11.7–65.6) when placed on the ankle, demonstrating no significant differences during the week (Fig. 1). Regarding infants capable of walking, the mean acceleration was higher and very similar between placements (wrist: 28.3 (95% CI, 26.6–30.0); ankle: 28.4 (95% CI, 25.6–31.3)).

**TABLE 1:**
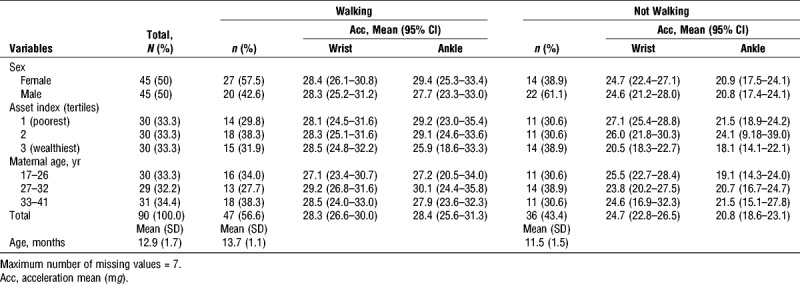
Socioeconomic characteristics and mean acceleration of the sample (Pelotas, RS, Brazil (2016)).

**FIGURE 2:**
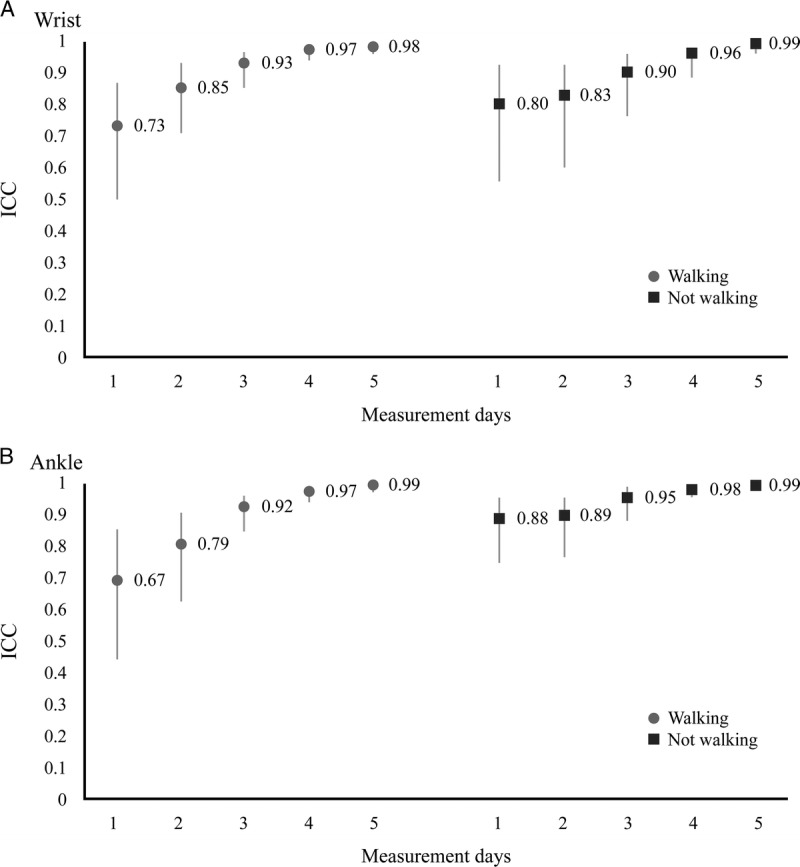
A, ICC and respective 95% CI comparing the mean acceleration of 1 to 5 measurement days and the mean acceleration of 6 measurement days, stratified by walking status with the accelerometer placed on the wrist. B, ICC and respective 95% CI comparing the mean acceleration of 1 to 5 measurement days and the mean acceleration of 6 measurement days, stratified by walking status with the accelerometer placed on the ankle.

Figures 2A and B present the ICC comparing the mean acceleration of 1 to 5 d of measurement and the mean acceleration of 6 measurement days, stratified by walking status, with the accelerometer placed on the wrist and ankle. In general, infants incapable of walking showed less variability among days, achieving an ICC of 0.80 with just 1 d of measurement in both placements, whereas among those capable of walking, this threshold was obtained within 2 d for the wrist (0.85; 95% CI, 0.71–0.93) and 3 d for the ankle placement (0.92; 95% CI, 0.84–0.96). Concerning the subgroup analysis (see Table, Supplemental Digital Content 10, ICC of the comparison between different numbers of measurement days and the standard of 6 complete days of measurement stratified by walking status among infants using the accelerometer on the wrist, http://links.lww.com/MSS/B125), for the wrist placement, the ICC threshold greater than 0.8 was reached within at least 2 d of measurement for all subgroups, except for those classified in the first tertile of the asset index among infants incapable of walking, who reached the threshold within 4 d (0.81; 95% CI, 0.36–0.96), and also the first category of maternal age (17–26 yr) where the threshold was reached within 3 d of measurement (0.88; 95% CI, 0.50–0.98).

In the group with the ankle placement of the device (Fig. 2B), there was greater variability among the results than that found with the wrist analyses. Among infants capable of walking, the threshold of 0.8 was reached within 3 d of measurement for boys (0.83; 95% CI, 0.53–0.95). Furthermore, in the subgroup analysis, the poorest economic group (0.87; 95% CI, 0.51–0.97) and those with mothers age between 33 and 41 yr (0.92; 95% CI, 0.71–0.98) showed the same pattern. The remaining groups presented an ICC of 0.8 within 2 d of measurement (see Table, Supplemental Digital Content 11, ICC of the comparison between different numbers of measurement days and the standard of 6 complete days of measurement, stratified by walking status among infants using the accelerometer on the ankle, http://links.lww.com/MSS/B126). For infants unable to walk, 1 d of measurement was capable of accurately representing the 6-d measurement mean acceleration with an ICC greater than 0.8.

**FIGURE 3:**
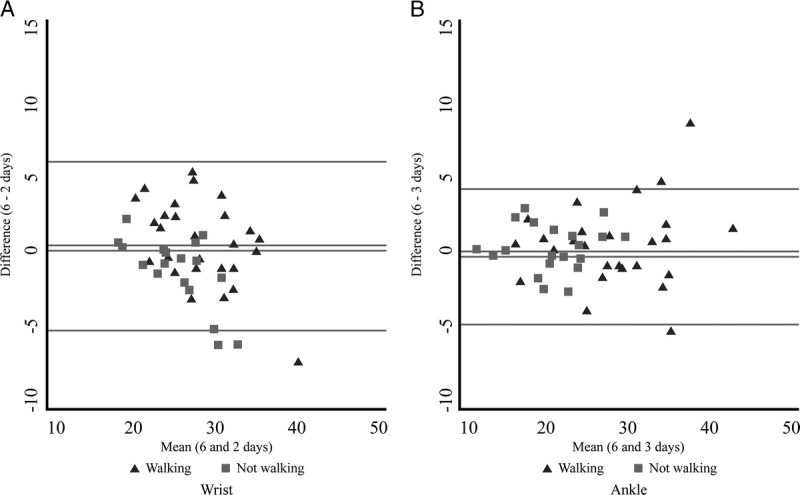
A, Bland–Altman plot of the difference between the mean acceleration of 6 and 2 measurement days with the accelerometer placed on the wrist. B, Bland–Altman plot of the difference between the mean acceleration of 6 and 3 measurement days with the accelerometer placed on the ankle.

Figures 3A and B present the Bland–Altman plot comparing the criterion measure (6 d) and the minimum measurement days of each placement, as shown previously. Overall, there was good agreement between the means for 2 and 6 measurement days on the wrist (mean bias, 0.39 m*g*). However, the difference between the acceleration for 2 and 6 d is positively correlated with the bias, indicating that the use of 2 measurement days tends to overestimate the mean acceleration for those with higher values.

For the ankle placement, the results showed higher variability, especially among walking infants; there was also a relatively small difference between the 6 and 3 measurement days (mean bias, 0.34 m*g*). In general, among infants capable of walking, there was higher variability and therefore less agreement between the periods in comparison with those incapable of walking. The comparisons between the remaining periods of measurement are shown in the Supplemental Digital Content. As expected, the agreement between periods tends to improve with the increase of measurement days, and there is lower variability in the analyses restricted to infants incapable of walking (see Figures, Supplemental Digital Content 2–9, http://links.lww.com/MSS/B117, http://links.lww.com/MSS/B118, http://links.lww.com/MSS/B119, http://links.lww.com/MSS/B120, http://links.lww.com/MSS/B121, http://links.lww.com/MSS/B122, http://links.lww.com/MSS/B123, http://links.lww.com/MSS/B124).

**FIGURE 4:**
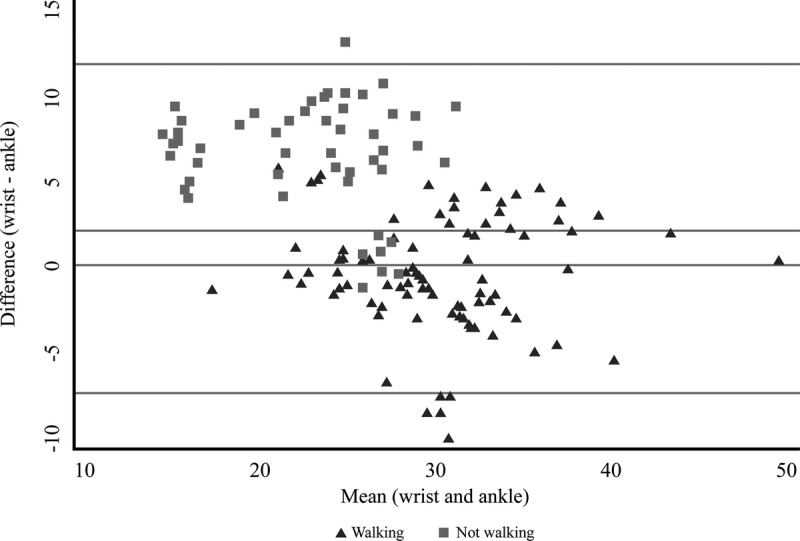
Bland–Altman plot of the difference between the mean acceleration of 6 measurement days with the accelerometer placed on the wrist and on the ankle among infants who used the device in both placements.

Furthermore, Figure 4 shows the Bland–Altman plot comparing wrist and ankle placement regarding the 6-d mean acceleration, restricted for infants who used the device in both placements simultaneously, stratified by walking status. The limits of agreement show a mean difference of 2 m*g* between the two placements (7% of the mean), demonstrating a good agreement when looking at the overall sample. However, when stratifying for walking status, the wrist placement shows a higher acceleration in comparison with the ankle for those infants who did not walk (mean bias, 6.75 m*g*) and a small difference between placements among those already walking (mean bias, −0.90).

#### Qualitative approach

For the qualitative section, 89 mothers/caregivers were interviewed regarding relevant research questions for the establishment of a measurement protocol for infants’ physical activity using accelerometry. One mother/caregiver was not available for interview and was considered as a loss. The interview script and relevant responses for each placement group (wrist, ankle, both) are available in the Supplemental Digital Content (see Tables, Supplemental Digital Content 12, Interview script given to the infant’s mothers or guardians, http://links.lww.com/MSS/B127; and Table, Supplemental Digital Content 13, Main interview topics and common responses for each placement group (wrist, ankle, both), http://links.lww.com/MSS/B128). The analyses of the overall interviews demonstrated that the routine of most children seemed to be similar throughout the week. This was particularly true among those attending daycare, where the timetable of meals, naps, and activities was maintained in almost the same way every day. In addition, the infants seemed to prefer having a fixed routine; some mothers reported noticing irritation and sadness when something changed in the infants’ daily routine. This evidence supports the hypothesis that a smaller number of measurement days could be adequate to represent physical activity for children in this age range.

Another topic of interest in the interviews was the reaction and potential changes on the infants’ behavior during the time of using the device. Most mothers reported an irritability or discomfort in the first day of use and indifference in the following days. The common description was “He/she didn’t even notice the accelerometer.” However some mothers related that there were sleep problems or too much crying during the period, especially the younger mothers and those with higher socioeconomic status.

Regarding difficulties for the mothers’ during the measurement period, there was a general complaint related to dressing the child, because clothes for infants are usually tight in the wrist and ankle, especially for girls who often wear pantyhose and tight-fitting outfits, making the process more difficult than usual. One mother even reported wanting to take off the accelerometer because of the dressing difficulties.

Despite the fact that most mothers did not have any concerns, some were worried that the device could be too tight and might hurt the baby’s skin. Regarding compliance, 10 mothers removed the accelerometer before the end of 7 d, but this did not differ by placement (five withdrawals on each). Mothers of higher socioeconomic status were the ones with greater fear of using the device, and also the group that had most device withdrawals. The accelerometer’s size was frequently mentioned as a concern, because it is relatively big for children in this age group, and the large number of measurement days was mentioned as a problem. Some mothers pointed to the 7-d protocol as a reason for withdrawal of the device and stated that if the period proposed had been shorter, they would not have removed the accelerometer.

The fundamental question of the interview was which was the preferred location, wrist or ankle, for wearing the accelerometer. The response was especially important from the group wearing the device in both placements, whose mothers/caregivers were able to visualize the pros and cons of each placement simultaneously. Despite the initial concerns, the wrist placement was the preferred location among the overall sample; however, among infants tested in both placements, exactly half of the mothers chose the wrist. The positive answers regarding the ankle placement were based mainly on the ease of dressing the child and the visibility of the accelerometer. Furthermore, mothers reported feeling more discomfort when the device was placed on the ankle, mainly because the device stays hidden by the clothes, making it difficult to observe any indication of possible injuries.

## DISCUSSION

The present study was designed to establish a protocol for measuring physical activity by accelerometry in infants age approximately 12 months. To our knowledge, there has been little research focusing on measuring physical activity on such a young sample, hence the need to test the best measurement protocol considering data quality as well as safety and comfort for the participants.

First, our sample showed potential reactivity to the device in the first day of use. Although day 1 had a shorter period of measurement, there were higher activity levels than during the remaining days, although it would be expected to present lower activity levels. The responses from the qualitative interviews also demonstrated that most infants presented irritability or discomfort in the first day of use and indifference in the following days. These findings could serve as a recommendation for future researchers to pay attention to this possibility and to take this into account when defining the study protocols to allow the infant to get used to the device before starting data collection, therefore avoiding overestimations or bias.

We took an ICC of 0.80 as a threshold for reliability between the criterion measure of 6 d and the remaining measurement days. When looking at the overall sample, we found that 2 d was sufficient, regardless of walking status, when the accelerometer was placed on the wrist; 3 d was sufficient when the accelerometer was placed on the ankle; and for infants incapable of walking, just 1 d was sufficient to represent the criterion measure, due to less variability among days. However, it is imperative to highlight that longer periods of measurement result in more precise measures, especially for infants already walking, who present greater acceleration variability throughout the week. The 95% limits of agreement (presented in smallest detectable change, 2–9) were reduced when 4 and 5 measurement days was assessed, decreasing the amount of error that may be introduced by using shorter measurement periods. In this sense, decisions regarding the minimum number of days of measurement must seek a balance between measurement reliability and precision and the participants’ comfort and acceptability of the device.

Our results showed a lower minimum days of measurement compared with previous research, although it is important to emphasize that the literature available refers to older children. Kang et al. (21) demonstrated that 4 d with an ankle-worn accelerometer (StepWatchTM) is sufficient to represent 7 d of step count monitoring among 2- to 3-yr-old North American children. The study of Hislop et al. (22) established that a minimum of 3 d of accelerometry monitoring, regardless of whether it included a weekend day, for at least 7 h daily, offers sufficient reliability to characterize total physical activity of preschool Scottish children (mean age, 3.7 yr) using uniaxial GT1M and GT3X accelerometers (ActiGraph, Walton Beach, FL). Similarly, Bingham et al. (23) found that, in a 7-d protocol wearing GT3X accelerometers (ActiGraph), for at least 6 h in any 3 d of a week, demonstrated good reliability when analyzing counts-based data among preschoolers (2.93 ± 0.59 yr).

The parameters used to determine the minimum number of measurement days and best placement of the device in the present study were based both on quantitative and on qualitative data; in addition, the knowledge of motor characteristics of this age range needs to be considered. On this topic, the first year of life is marked by a variety of movements—such as rolling, sitting, lifting, feeding, and finally walking—increasing manipulative and locomotion coordination (24). From 5 to 12 months, the infant makes several efforts toward walking, but each child has different processes for motor skills acquisition, parallel to cognitive development, and is susceptible to environmental influences (5). For this reason, it is possible for a healthy infant not to be capable of walking at 12 months, depending on several processes and stimuli that may or may not have occurred in this period (25). This is the main reason why the stratification of physical activity data by walking status is important, because within a sample composed of infants all the same age, there may be differences in the motor development, which reflect directly on the acceleration captured by the device. This difference is illustrated in the present study by the lower variation and greater agreement between days found among infants incapable of walking in comparison with infants already capable of walking.

Despite this issue, the choice that brings together a lower time of exposure to the device, good agreement in comparison with the 6-d measurement, and a good acceptability among mothers and infants was the wrist placement. The use of the accelerometer on the wrist is a trend reported in the accelerometry literature, mainly because of the greater compliance to the protocol, besides the possibility of evaluation of sleep duration and quality. The National Health and Nutrition Examination Survey, a study considered a reference in the area of physical activity measurement worldwide, modified the measurement protocol for use with the wrist, hoping to improve compliance of the participants (26). Furthermore, it is important to maintain the same placement of the device during all follow-ups to enable future longitudinal comparisons.

Furthermore, our analyses comparing accelerometers’ placement were performed basically to gain insights regarding differences and similarities in the total amount of movement captured, to justify further analyses focused on the research protocol. Future studies must address further descriptions of movement patterns among toddlers. It seems that wrist and ankle placement provides a similar acceleration mean value among children who have already started to walk, although among those who cannot walk, the wrist placement showed a higher acceleration mean compared with the ankle. This result could be expected because of the activity patterns of infants who are still making efforts toward walking; for example, seated activities with intense movement of upper limbs and trunk are common, especially in the exploration of objects (27). In these activities, movements would be captured by the accelerometer on the wrist, but lower levels of acceleration signals would be captured if the accelerometer was placed on the ankle.

Regarding the qualitative interviews, the responses showed a good acceptability of the accelerometer in both placements, yet the wrist placement was the preferred among the overall sample. Also, mothers with higher socioeconomic status were more concerned regarding the device use. A Belgian study aiming to verify the feasibility and validity of accelerometer measurements among 47 toddlers (1- to 3-yr-olds), wearing a GT1M (ActiGraph) accelerometer fixed on the waist for 6 consecutive days, showed that 83% of the parents perceived wearing the accelerometer as “not unpleasant and not pleasant,” whereas none perceived it as “unpleasant” (4). Furthermore, a qualitative study used focus group meetings with 17 South Asian and white British mothers and fathers of 2- to 3-yr-old children to assess the qualitative feasibility and acceptability of using three different accelerometers placed on the waist; their results showed the ActiGraph GT3X as the preferred device for both children and parents (28). There were no studies available verifying the acceptability of the accelerometer comparing different body locations among infants, and the available literature focused on waist placement and older children. It is our understanding that there are some obstacles related to waist-worn accelerometers on young children, such as the large amount of sitting and playing activities, when the waist placement may not adequately quantify the infants’ activities; in addition, the use of diapers and the process of clothing could be considered as a barrier. Further research adding direct observation and qualitative interviews to the accelerometry data is needed to better understand the feasibility of different placements among infants.

Finally, to the best of our knowledge, this is the first study in developing countries to access the best methods of measurement and acceptability of the accelerometer among young children. The use of raw data is also an important strength of the study, taking a step toward future comparability between studies regarding data processing. Also, the present study tries to bring together different methodological approaches to better understand the measurement issues regarding this young sample. However, some limitations need to be considered, such as the small sample size for stratified analyses, which resulted in wide CI among some subgroups. Furthermore, the convenience sampling may affect the representativeness of our results, although we believe that at this age range, the behavior and habits of the infants are similar, and our sampling process gathered different subgroups of the population, especially regarding socioeconomic status.

## CONCLUSIONS

On the basis of our results, among infants between 9 and 15 months of age, 2 and 3 measurement days with the accelerometer placed on the wrist and ankle, respectively, seemed to be representative of a week of measurement. An accelerometer placed at the wrist had better acceptance by the infants and mothers. We emphasize that reactivity to the device in the first few hours is possible; therefore, we recommend that researchers program the accelerometer for beginning data collection at midnight, to allow the infant to get used to the device during the first day. Thus, around 3 d of accelerometer use could be recommended in a final study protocol; the first day would be to avoid reactivity and then 2 d for measurement.

## Supplementary Material

SUPPLEMENTARY MATERIAL
