# Supplementary material for: Protocol for Objective Measurement of Infants’ Physical Activity using Accelerometry
Source: Med Sci Sports Exerc. 2017 Dec 2;50(5):1084–92. doi: 10.1249/MSS.0000000000001512 (PMC5849301; doi:10.1249/MSS.0000000000001512)
Supplement: SUPPLEMENTARY MATERIAL [file mss-50-1084-s003.pdf]

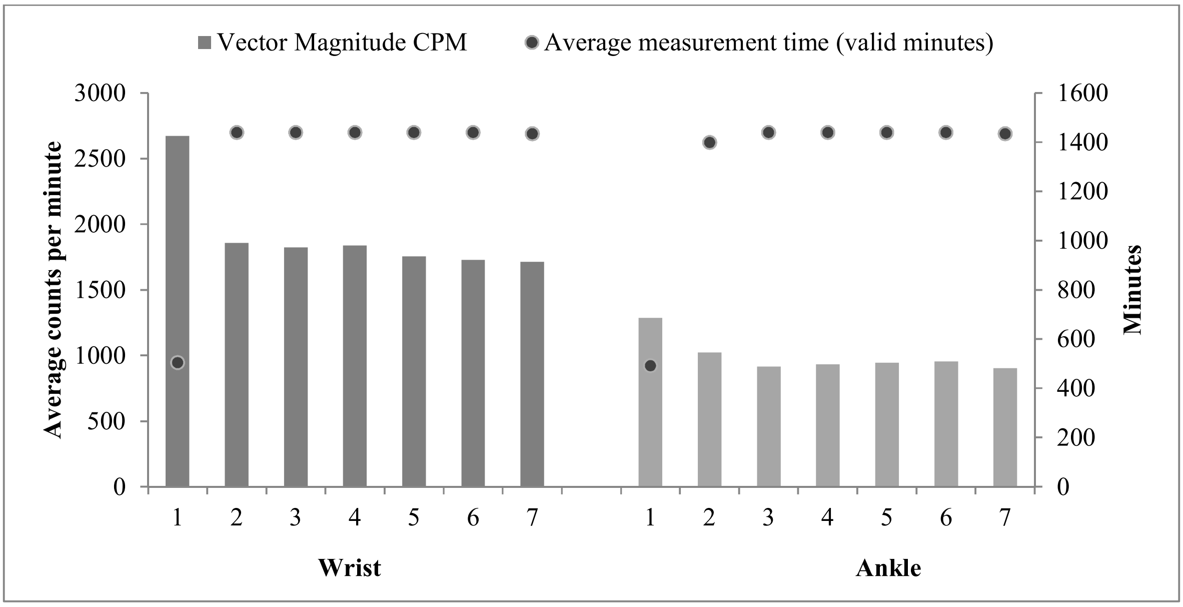

Supplemental Digital Content 1. Preliminary analysis presenting average daily Vector Magnitude counts per minute (CPM) and average measurement valid minutes in each placement of the accelerometer.

The illustration above presents a preliminary counts-based analysis, showing evidence of potential reactivity. The first day showed higher activity level in comparison with the remaining days, despite the shorter measurement period. We attributed this to reactivity because the infant may present higher activity while exploring and getting used to the device. This interpretation was reinforced based on mother's qualitative interview.
