# Supplementary material for: Protocol for Objective Measurement of Infants’ Physical Activity using Accelerometry
Source: Med Sci Sports Exerc. 2017 Dec 2;50(5):1084–92. doi: 10.1249/MSS.0000000000001512 (PMC5849301; doi:10.1249/MSS.0000000000001512)
Supplement: SUPPLEMENTARY MATERIAL [file mss-50-1084-s005.pdf]

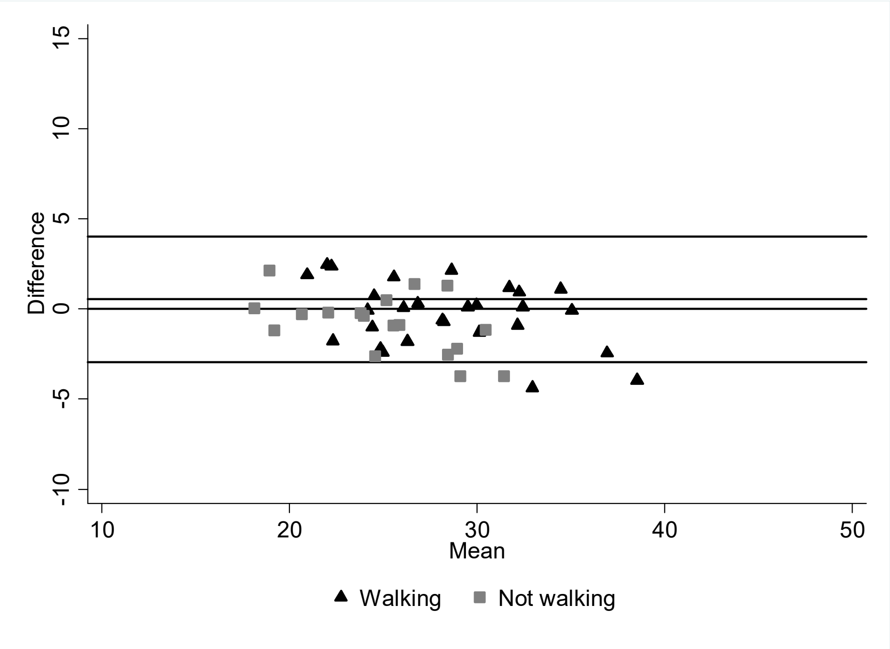

Supplemental Digital Content 3. Bland & Altman graph of the difference between the mean acceleration of 6 and 3 measurement days with the accelerometer placed on the wrist.
