# Supplementary material for: Protocol for Objective Measurement of Infants’ Physical Activity using Accelerometry
Source: Med Sci Sports Exerc. 2017 Dec 2;50(5):1084–92. doi: 10.1249/MSS.0000000000001512 (PMC5849301; doi:10.1249/MSS.0000000000001512)
Supplement: SUPPLEMENTARY MATERIAL [file mss-50-1084-s012.pdf]

Supplemental Digital Content 10. Intraclass correlation coefficient of the comparison between different numbers of measurement days and the standard of six complete days of measurement stratified by walking status among infants using the accelerometer on the wrist.

		Intraclass Correlation Coefficient (CI 95%)*				
**Variables**	**N**	**1 day**	**2 days**	**3 days**	**4 days**	**5 days**
Walking						
Sex						
Female	12	0.76 (0.47; 0.91)	0.83 (0.58; 0.93)	0.92 (0.78; 0.97)	0.97 (0.91; 0.99)	0.98 (0.95; 0.99)
Male	16	0.69 (0.24; 0.89)	0.91 (0.73; 0.97)	0.96 (0.87; 0.99)	0.97 (0.92; 0.99)	0.98 (0.94; 0.99)
Asset index (tertiles)						
1 *(poorest)*	9	0.64 (0.05; 0.90)	0.87 (0.57; 0.97)	0.96 (0.83; 0.99)	0.96 (0.83; 0.99)	0.96 (0.86; 0.99)
2	11	0.77 (0.38; 0.93)	0.85 (0.57; 0.96)	0.94 (0.79; 0.98)	0.98 (0.92; 0.99)	0.99 (0.97; 1.0)
3 *(richest)*	8	0.75 (0.21; 0.94)	0.86 (0.49; 0.97)	0.91 (0.65; 0.98)	0.98 (0.91; 1.0)	0.99 (0.98; 1.0)
Maternal age (years)						
17 to 26	11	0.78 (0.39; 0.93)	0.89 (0.67 (0.97)	0.97 (0.89; 0.99)	0.97 (0.89; 0.99)	0.98 (0.94; 1.0)
27 to 32	8	0.52 (-0.17; 0.88)	0.84 (0.43; 0.96)	0.94 (0.77; 0.99)	0.98 (0.90; 1.0)	0.96 (0.84; 0.99)
33 to 41	9	0.78 (0.32; 0.94)	0.85 (0.50; 0.96)	0.90 (0.66; 0.98)	0.97 (0.89; 0.99)	0.99 (0.97; 0.99)
Not walking						
Sex						
Female	9	0.75 (0.26; 0.93)	0.93 (0.73; 0.98)	0.93 (0.74; 0.98)	0.95 (0.82; 0.99)	0.99 (0.95; 1.0)
Male	9	0.84 (0.46; 0.96)	0.81 (0.40; 0.95)	0.90 (0.66; 0.98)	0.96 (0.85; 0.99)	0.98 (0.94; 1.0)
Asset index (tertiles)						
1 *(poorest)*	8	0.38 (-0.33; 0.83)	0.33 (-0.39; 0.81)	0.53 (-0.16; 0.88)	0.81 (0.36; 0.96)	0.93 (0.73; 0.99)
