# Supplementary material for: Protocol for Objective Measurement of Infants’ Physical Activity using Accelerometry
Source: Med Sci Sports Exerc. 2017 Dec 2;50(5):1084–92. doi: 10.1249/MSS.0000000000001512 (PMC5849301; doi:10.1249/MSS.0000000000001512)
Supplement: SUPPLEMENTARY MATERIAL [file mss-50-1084-s014.pdf]

Supplemental Digital Content 12. Interview script applied to infant’s mothers/guardians.

FEDERAL UNIVERSITY OF PELOTAS

POST GRADUATION PROGRAM IN EPIDEMIOLOGY

Protocol study – Accelerometry among infants

Interview script

We would like to talk with you (mother/guardian) about the period which your child wore the accelerometer. It is very important that you tell us everything that happened honestly and in details. It is quite an informal conversation, but of most value for my PhD thesis. This device will be used by a great number of children, and your help is of great importance! Your opinions and experiences, good or bad, are very valuable. I’ll start with one question and we will go on talking. Ok?

1) Tell me about <name of the infant>’s daily routine. How he/she plays? Is he/she already walking? Where he/she spends the day and who takes care of him/her?
2) How was the health and mood of your son/daughter in the days wearing the accelerometer?
3) How was your son’s/daughter’s reaction regarding the device?
4) Did you notice any difference in his/her behavior during this period?
5) Wearing the accelerometer modified your daily routine in any way? Did you have any difficulties?
6) Which of the two accelerometers were more comfortable for you and your son/daughter? <only for the “wrist + ankle” group>
7) Do you think it would be better if the device were worn in other location (wrist or ankle)? <for the “only wrist” or “only ankle” groups>
8) Did you worry about the possibility of your son/daughter getting hurt or injured? <Verify why and where she worried>
9) Did you wanted to take the device off? Why? <Explore what made her take it off, which device (for the wrist + ankle group)
10) How was the drying process after shower or pool bath? <Explore if any contact dermatitis were perceived>
11) If we hypothetically as your son/daughter to wear the accelerometer for a few days longer, would you accept?
12) Do you wish to report anything else or do you have any advice for us?

Thank you for your help!
