# Supplementary material for: Protocol for Objective Measurement of Infants’ Physical Activity using Accelerometry
Source: Med Sci Sports Exerc. 2017 Dec 2;50(5):1084–92. doi: 10.1249/MSS.0000000000001512 (PMC5849301; doi:10.1249/MSS.0000000000001512)
Supplement: SUPPLEMENTARY MATERIAL [file mss-50-1084-s015.pdf]

Supplemental Digital Content 13. Main interview topics and common responses for each placement group (wrist, ankle, both).

Main discussion topics	Placement	Quotes
Infant's reaction	Wrist	"In the first day he tried to remove it, he kept starring at it ... After that he started acting normal, he got used to it, didn't touched it any longer"
	Ankle	"It was normal, he didn't even notice ... Only in the shower, he would play with it (the device), but soon he forgets about it..."
	Both	"In the day (first) she tried to eat it, put them on her mouth.. But after ... I thought she would try to take them off, but it surprised me"
Behavior changes	Wrist	"She didn't even mind it"
	Ankle	"No, I do not think he even noticed that he had a device"
	Both	"No, normal... As usual"
Routine changes/Difficulties	Wrist	"Troubles a little to dress, but no big deal"
	Ankle	"No, because boys are easier, boys have larger clothes ... Girls would be more complicated... leggings"
	Both	"Some clothes were not so good to wear ... the one of the foot (ankle) when I tried tighter pants, the socks for example I couldn't put it on top, I had to put underneath (the accelerometer)... I think it would have to be smaller"
Concerns	Wrist	"Just because of the size.. fear that could cause some kind of allergy, that could be tightening... this was more on the first day, as she was crying I thought maybe it is too tight... and we were advise not to take it off..."
	Ankle	"I got concern at night, that he could hurt himself...but it didn't happened"
	Both	"My concern is if she was going to feel something, like an electric shock"
Wished to remove the device	Wrist	"No, it didn't bothered her"
	Ankle	"No, it didn't come to that"
	Both	"When we went out... It was ugly... but then I covered it with clothes"

Best placement	Wrist	"I believe the ankle is more difficult, but it wouldn't change much"
	Ankle	"I think he would play more with it, he has more access to the arm than to the ankle ... I found interesting in the ankle"
	Both	"The one of the hand {(father) it was not to be} ... he forgot about it ... the one of the leg was very big. Here (in the wrist) we use as a watch, but there (ankle) we put socks, we put boots, put a lot of things on top, it gets too tight..."
